# Supplementary material for: Splice-Junction-Based Mapping of Alternative Isoforms in the Human Proteome
Source: Cell Rep. Author manuscript; Available in PMC 2020 Jan 15. (PMC6961840; doi:10.1016/j.celrep.2019.11.026)
Supplement: 2 [file NIHMS1546469-supplement-2.zip › DF1/PXD006675/RightVentricle/RightVentricle_27_GSN_AELAMEKLFCCFPNSMVVEHPEFLK.pdf]

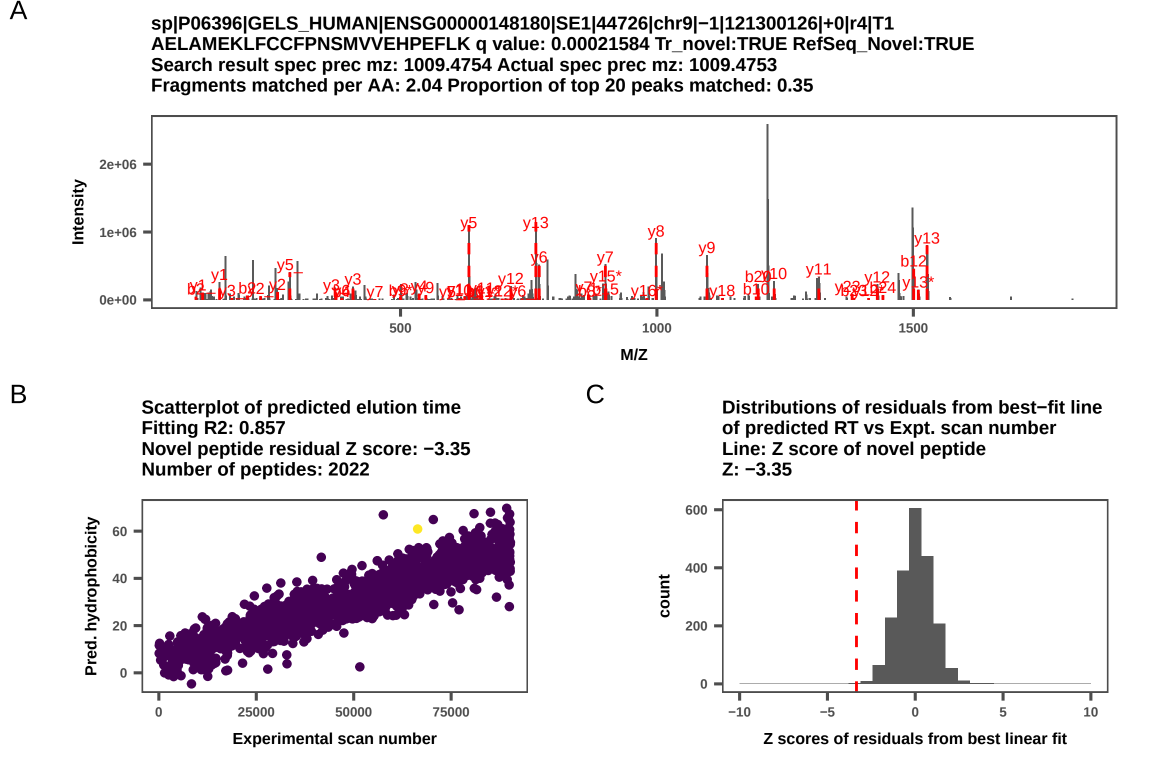
A
sp|P06396|GELS_HUMAN|ENSG00000148180|SE1|44726|chr9|−1|121300126|+0|r4|T1
AELAMEKLFCCFPNSMVVEHPEFLK q value: 0.00021584 Tr_novel:TRUE RefSeq_Novel:TRUE
Search result spec prec mz: 1009.4754 Actual spec prec mz: 1009.4753
Fragments matched per AA: 2.04 Proportion of top 20 peaks matched: 0.35
Intensity
2e+06
1e+06
0e+00
500
1000
1500
M/Z
y5
y13
y8
y9
y6
y7
y15*
b20
y10
y11
y13
b12
y12
y18
B
Scatterplot of predicted elution time
Fitting R2: 0.857
Novel peptide residual Z score: −3.35
Number of peptides: 2022
Pred. hydrophobicity
60
40
20
0
0
25000
50000
75000
Experimental scan number
C
Distributions of residuals from best−fit line
of predicted RT vs Expt. scan number
Line: Z score of novel peptide
Z: −3.35
count
600
400
200
0
−10
−5
0
5
10
Z scores of residuals from best linear fit
